# Very low calorie ketogenic diet combined with physical interval training for preserving muscle mass during weight loss in sarcopenic obesity: A pilot study

**DOI:** 10.3389/fnut.2022.955024

**Published:** 2022-09-29

**Authors:** Elisabetta Camajani, Alessandra Feraco, Stefania Proietti, Sabrina Basciani, Luigi Barrea, Andrea Armani, Mauro Lombardo, Lucio Gnessi, Massimiliano Caprio

**Affiliations:** ^1^Department of Experimental Medicine, Sapienza University of Rome, Rome, Italy; ^2^Department of Human Sciences and Promotion of the Quality of Life, San Raffaele Roma Open University, Rome, Italy; ^3^Laboratory of Cardiovascular Endocrinology, Istituto di Ricovero e Cura a Carattere Scientifico (IRCCS) San Raffaele, Rome, Italy; ^4^Unit of Clinical and Molecular Epidemiology, Istituto di Ricovero e Cura a Carattere Scientifico (IRCCS) San Raffaele, Rome, Italy; ^5^Dipartimento di Scienze Umanistiche, Università Telematica Pegaso, Naples, Italy; ^6^Department of Clinical Medicine and Surgery, Endocrinology Unit, Centro Italiano per la cura e il Benessere del Paziente con Obesità, University Medical School of Naples, Naples, Italy

**Keywords:** VLCKD, sarcopenia, physical activity, fat free mass, fat mass

## Abstract

**Background:**

The prevalence of sarcopenic obesity (SO) is increasing worldwide, posing important challenges to public health and national health care system, especially during the COVID pandemic. In subjects with SO, it is essential to reduce body weight, and to preserve lean mass, to avoid worsening of muscle function. Adequate nutrition and correct physical activity is essential to counteract SO progression. Very Low Calorie Ketogenic Diet (VLCKD), a well-established nutritional intervention for obesity, has been also indicated for the treatment of SO. To date, the effects of physical training during VLCKD have not been investigated.

**Aim:**

This pilot study aims to determine the efficacy of VLCKD combined with interval training, compared to a VLCKD alone, on weight-loss, body composition, and physical performance in participants with SO.

**Materials and methods:**

Twenty-four participants with SO, aged between 50 and 70 years, who met the inclusion criteria, accepted to adhere to a VLCKD (<800 Kcal/die) and to give informed consent, were enrolled in the study. Twelve participants followed a structured VLCKD protocol (VLCKD group) and twelve followed the same VLCKD protocol combined with interval training (IT), twice per week (VLCKD + IT group). Data were collected at baseline (T0) and after 6-week of treatment (T6). Anthropometric indexes, body composition analysis by Bioelectrical Impedance Analysis (BIA), muscle strength and physical performance analysis were assessed at baseline and at the end of treatment.

**Results:**

At the end of the study, body mass index (BMI), body weight, waist circumference, and hip circumference were significantly reduced in both VLCKD group and VLCKD + IT group. Interestingly, a significant improvement in muscle strength and physical performance was observed in both groups. A multiple comparison of delta variations in all parameters between groups was performed. No differences were observed for the majority of anthropometric and biochemical parameters, with the exception of fat free mass (FFM) and fat mass (FM): notably, participants who followed a VLCKD combined with IT preserved FFM (*p* < 0.001) and reduced FM (*p* = 0.001) to a greater extent than what observed in VLCKD group. Moreover, high density lipoprotein (HDL) cholesterol plasma levels were significantly higher in the VLCKD + IT group compared to the VLCKD group.

**Conclusion:**

This pilot study confirms that VLCKD is effective in terms of body weight reduction, particularly FM; moreover, the combination of VLCKD and interval training could determine a better preservation of FFM.

## Introduction

The term “sarcopenia” was first coined by Rosenberg, initially referring only to age-related loss of muscle mass (1). In recent years, the literature has been referring to Sarcopenic Obesity (SO), which is a clinical condition characterized by an excess of fat mass (FM) and a reduction of muscle mass (2); as proposed by Barazzoni et al. (3), the term “sarcopenic obesity” has been used to describe obesity with reduced skeletal muscle function and mass. In a meta-analysis conducted by Hsu et al. (4), it was stressed that while the concept of SO is clear, its operational definition is still inconsistent. In 2022, the European Society for Clinical Nutrition and Metabolism (ESPEN) and the European Association for the Study of Obesity (EASO) launched an initiative to reach an expert consensus on the definition and diagnostic criteria of SO (5). As reported, a diagnosis of SO should be considered in high-risk individuals with concomitant elevated BMI or waist circumference (WC) and positive tests for markers of low skeletal muscle mass and function (e.g., clinical symptoms or validated questionnaires). Moreover, individuals with SO should be further stratified into stage I in the absence of clinical complications, or stage II if cases are associated with complications linked to altered body composition or skeletal muscle dysfunction.

Lifestyle interventions, such as calorie restriction and physical activity, represent the main strategies for prevention and treatment of SO, as they can prevent or slow down the pathophysiological processes underlying its development (6, 7). Ketogenic diet, in particular very low calorie ketogenic diet (VLCKD), is now recognized as an effective nutritional strategy, in particular during the COVID-19 pandemic (8, 9), to treat sarcopenic obesity (10). VLCKD are characterized by a daily caloric intake of 800 Kcal; from a macronutrient point of view, VLCKD provide, as stated in the Position Statement of the Italian Society of Endocrinology (SIE), 1.2–1.5 g of protein, 30 g of fat, derived from meal replacement and olive oil, and 30 g of carbohydrates, derived mainly from vegetables and to a small extent from meal replacement (11). Moreover, the use of digital platforms has been proven effective in treating patients who need medical and/or nutritional support, and in favoring dietary adherence and/or exercise-based interventions (12). Camajani et al. (13) recently described the efficacy of a combined approach intervention including a VLCKD, along with interval training (IT) in reducing FM, improving metabolic profile, and preserving skeletal muscle performance of a female subject after hospitalization for severe COVID-19. On the basis of these considerations, this pilot study aims to determine the efficacy of a VLCKD combined with physical interval training, compared to a VLCKD alone, on weight-loss, body composition, and physical performance, in patients with SO, to test the hypothesis that physical training during VLCKD can preserve free fat mass (FFM) better than VLCKD alone. The primary outcome was the reduction of total body weight (BW) and FM, and the preservation of free fat mass (FFM).

## Materials and methods

### Study design

This was an open, nutritional intervention, pilot study that enrolled participants with SO among those attending the Center for the Study of Eating Disorders and Obesity, Department of Experimental Medicine, Section of Medical Pathophysiology, Food Science, and Endocrinology of the University of Rome “La Sapienza,” Italy. This trial was registered at clinicaltrials.gov (NCT05287659).

### Inclusion and exclusion criteria

The inclusion criteria were as follows: women and men, age between 50 and 70 years old, BMI between 30 and 40 kg/m^2^ with stable BW in the previous 6 months, WC ≥ 102 cm for men and ≥88 cm for women, sarcopenia, insulin resistance (Homeostasis Model Assessment-Insulin Resistance, HOMA-IR ≥ 2.5) or type 2 diabetes mellitus treated only with metformin.

The presence of SO was considered when the following conditions were satisfied:

- FM > 39–41% for woman and > 29–31% for man, according to ESPEN and EASO Consensus Statement (5).
- Five times Sit-to-Stand Chair test > 15 s according to EWGOP2 (14).
- Short physical performance battery (SPPB) < 8, according to EWGSOP2 (14).

The exclusion criteria were the following conditions: lack of informed consent, hypersensitivity to components of meals replacement, type 1 diabetes; cell failure in type 2 diabetes mellitus, insulin therapy or concomitant use of sodium/glucose cotransporter 2 (SGLT2) inhibitors, gastrointestinal diseases; hydroelectrolytic alterations; psychiatric disturbances; pregnancy; lactation; kidney failure [estimated glomerular filtration rate (eGFR) < 60 mL/min]; liver failure; heart failure (NYHA III-IV); respiratory failure; planned surgeries; unstable angina or cardiac arrhythmias; recent stroke or myocardial infarction (<12 months) (11, 15).

### Anthropometric assessment

Body weight, height, systolic and diastolic blood pressure (BP), WC, and hip circumference (HC) were measured at T0 and at the end of the protocol. Anthropometric measurements were recorded after an overnight fast under resting conditions using calibrated equipment. BW was measured using a balance-beam scale (Seca GmbH & Co, Hamburg, Germany). WC was measured midway between the costal arch and the iliac crest and HC was measured at the symphysis-greater trochanter level to the closest 1 cm (16). Height was rounded to the closest 0.5 cm. BMI was calculated as weight divided by squared height in meters (kg/m^2^). Systolic and diastolic BP were measured using a mercury-gravity manometer.

### Blood and urine chemistry

In accordance with the SIE Position Statement of the Caprio et al. (11), blood tests [blood count, electrolytes, glucose, insulin, lipids, total proteins and albumin, plasma creatinine, blood urea nitrogen (BUN) and uric acid, alanine transferase, aspartate transaminase, and Vitamin D levels] were performed before starting the protocol and after 6 weeks of diet therapy with meal replacements. Insulin resistance was determined using HOMA-IR (17). Estimation of the concentration of low-density lipoprotein cholesterol in plasma, without use of the preparative ultracentrifuge was determined at the beginning and at the end of the protocol (18). eGFR was calculated with Modification of Diet in Renal Disease (MDRD) (19). β-Hydroxybutyrate capillary blood levels were tested at the beginning of the study, every 2 weeks and at the end of the study, through a portable glucometer (GlucoMen LX Sensor, A. Menarini Diagnostics, Neuss, Germany; sensitivity < 0.2 mmol/L). The threshold value for nutritional ketosis was set at 0.5 mmol/L (20).

### Body composition

Body composition was measured through multifrequency Bioelectrical Impedance Analysis (BIA, Human Im Touch, DS Medica, Milan, Italy) at baseline and after 6 weeks (12). The Human Im Touch device records impedance at five frequencies (5, 10, 50, 100, and 250 kHz). During BIA, patients were lying supine. All measurements were performed on the patient’s right side. The four-surface standard tetra polar electrodes technique on the foot and hand was used.

### Muscle strength and physical performance analysis

To evaluate strength, chair stand test (CST) was performed, as indicated by the ESWGOP2 report (14). CST measured the amount of time needed for a patient to rise five times from a seated position without using arms: sarcopenia was diagnosed when the patient took more than 15 s to complete the task, as indicated by EWGSOP2 (14). To evaluate physical performance, the SPPB battery was used, consisting of three components of physical performance: (1) standing balance, (2) chair stands, and (3) gait speed. A score from 0 (poorest) to 4 (best) was assigned for each of these three components. The sum of the scores provided a composite score ranging from 0 to 12; physical performance was considered impaired when the total SPPB score was ≤ 8 (14).

### Dietary intervention

All patients followed a VLCKD [780–800 kcal/day, with the following composition in macronutrients, percentage of caloric intake, and g/kg of ideal BW of proteins (derived by the BMI set at 25 kg/m^2^), respectively: carbohydrates 26 g (13.5%), fats 35 g (40.4%) and proteins 80–90 g (1.2–1.4 g/Kg of ideal BW) for 6 weeks] (21). Patients were given four or five meals/day [timing was at main meals (7.30 a.m., 1.00 p.m., and 8.30 p.m.), mid-morning and mid-afternoon]. Supplements of vitamins, minerals and omega-3 fatty acids, were provided in accordance to international recommendations (22). The dietary fat component mainly derived from extra-virgin olive oil (20 g *per* day); in particular, polyunsaturated fatty acids (PUFA) proportion was 8%, monounsaturated fatty acid (MUFA) 77%, and the saturated fats 15%. The amount of daily fiber intake was about 25 g/day, as requested from Italian Guidelines (LARN 2014), mostly deriving from the vegetable servings. Participants received counseling by physicians and nutrition experts at baseline (T0) and every 2 weeks up to the end of the protocol (T6); dietary compliance was also assessed with a food frequency questionnaire, every 2 weeks. It was also recommended to drink not less than 2.0–2.5 L of water *per* day.

### Physical training intervention

Seven days after the beginning of the nutritional protocol, the patients enrolled in the VLCKD + IT group started Interval Training (IT) twice a week, with 48–72 recovery hours between sessions (13). Due to the pandemic, physical exercise sessions were carried out *via* Zoom platform with a personal trainer and each session lasted 30–35 min. Each session of physical exercise was structured as follows: an initial part of warm up, breathing and stretching, a second part based on functional exercises repeated for 20 s with a 10 s pause, a part of proprioception and balance, and finally a part focused on breathing. The required home-based equipment consisted of a chair with a backrest and without armrests, two bottles of water and a towel. Functional exercises proposed were changed every week; timing schedule was also modified, with an increase in exercise time and a decrease in recovery time. An example of the training session can be found in Table 1.

**TABLE 1:** Example of training session performed by patients of VLCKD + IT group.

Week 2—Day 1
3′ of breathing
3′ of warming up
Bird dog: Three series, 30” per side
Interval training:
**Round 1:** Partial squat with sitting on the chair (20 s of exercise—10 s of rest) Step up on rise (20 s of exercise—10 s of rest): Bird dog (20 s of exercise—10 s of rest): Lateral arm raises with 1.5 Kg (20 s of exercise) 30 s of rest **Round 2:** Partial squat with sitting on the chair (20 s of exercise—10 s of rest) Step up on rise (20 s of exercise—10 s of rest): Bird dog (20 s of exercise—10 s of rest): Lateral arm raises with 1.5 Kg (20 s of exercise) 30 s of rest **Round 3:** Partial squat with sitting on the chair (20 s of exercise—10 s of rest) Step up on rise (20 s of exercise—10 s of rest): Bird dog (20 s of exercise—10 s of rest): Lateral arm raises with 1.5Kg (20 s of exercise) 30 s of rest
3′ of stretching
3′ of breathing

### Data management and statistical methods

Variables were tested for normality of distribution using the Kolmogorov-Smirnov test. When non-normally distributed we used Wilcoxon test. The number of participants was identified considering the number of participants generally included in similar published pilot studies.

The demographics and clinical characteristics within groups were compared by paired Student’s *t*-test [data are expressed as mean values ± standard deviation (SD) for normally distributed variables] or Wilcoxon test (data are expressed as median values and interquartile range for non-normally distributed variables). Categorical variables were tested using the chi-square test or Fisher test (*n* < 5).

Between group differences were assessed by unpaired Student’s *t*-test (data are expressed as mean values ± SD for normally distributed variables) or Mann Whitney (data are expressed as median values and interquartile range for non-normally distributed variables).

Multiple and multivariate linear regression analyses were used to evaluate associations between the change in basal metabolic rate (BMR) and in body cell mass (BCM) respect to the change in FFM and FM into group, adjusting for age and sex.

Differences were considered statistically significant when *P* was ≤ 0.05.

Assuming a power of 90% and alpha of 0.05, 24 participants (total sample size, 12 participants in each of two groups) were considered appropriate to detect statistically significant differences in FM and FFM between treatment groups. Figures were performed usingx GraphPad Prism Version 6.00 for Windows, GraphPad Software, San Diego, California, USA. Statistical analysis was carried out with the statistical package SPSS 27.0 (SPSS, Inc., Chicago, IL, USA).

### Ethical aspects

The study protocol was approved by the Ethics Committee of the University of Rome “La Sapienza” (code 3920) and was conducted in accordance with the Declaration of Helsinki and Good Clinical Practice. All patients were informed about the possible risks and benefits of the proposed interventions and provided written consent.

## Results

**FIGURE 1:**
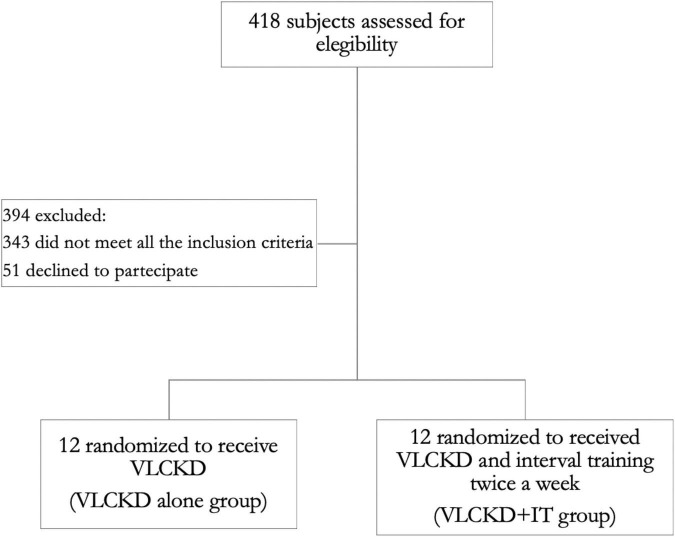
Flow diagram of the study. A total of 418 individuals were screened. The participants enrolled were randomized to a very low calorie ketogenic diet (VLCKD) dietary intervention group alone (VLCKD group) or a VLCKD dietary intervention group with interval training (VLCKD + IT group).

We screened 418 participants for eligibility; 394 were excluded (343 did not meet all the inclusion criteria and 51 declined to participate). We enrolled and randomized 24 participants from November 2021 to March 2022. Twelve patients underwent a VLCKD (VLCKD group) and 12 a VLCKD combined with interval training (VLCKD + IT group), as reported in Figure 1. All the participants were followed up to the completion of the study. Baseline characteristics of the patients were similar between groups and are summarized in Table 2. Adherence to the protocol was comparable between groups.

**TABLE 2:** Participants characteristics at baseline (T0).

	All	VLCKD	VLCKD + IT	*P*-value
Patients, *N* (%)	24	12 (50%)	12 (50%)	
**Sex, *N* (%)**				
M	3 (12.5)	2 (16.7)	1 (8.3)	0.537
F	21 (87.5)	10 (83.3)	11 (91.7)	
Age (years)	56.3 ± 5.3	56.0 ± 6.7	56.5 ± 3.8	0.795
Weight (Kg)	91.1 ± 9.8	90.6 ± 7.2	91.6 ± 12.2	0.810
BMI (kg/m^2^)	33.9 ± 3.4	33.9 ± 3.0	33.9 ± 3.8	0.973
HOMA-IR ≥ 2.5, *N* (%)	24 (100%)	12 (50%)	12 (50%)	1.000
FM, median (IQR)	36.36 (32.0–47.5)	35.90 (32.2–47.2)	40.02 (31.6–47.6)	0.887^+^
FFM (Kg)	52.4 ± 5.1	52.6 ± 5.4	52.2 ± 5.0	0.830
**Comorbidity**				
Type 2 diabetes mellitus	2	1	1	1.000
Hypertension	24	12	12	1.000
Hypercholesterolemia	16	7	9	0.386

Variables with normal distribution are expressed as mean ± SD, those with non-normal distribution as median (interquartile range) with Mann Whitney. VLCKD, very low calorie ketogenic diet; IT, interval training; BMI, body mass index; HOMA-IR, homeostatic model assessment for insulin resistance; FM, fat mass; IQR, interquartile range; FFM, fat free mass. ^+^Mann Whitney.

In summary, 22 of the 24 patients had insulin resistance, two had type 2 diabetes mellitus and were on metformin therapy. Eight patients were on antihypertensive therapy; 16 were dyslipidemic and only two were on statin treatment. 16 patients had a diagnosis of metabolic syndrome according to the NCEP ATP III diagnostic criteria (23). At the end of the study, two patients reduced the dose of metformin therapy, seven reduced or discontinued antihypertensive therapy, and one stopped lipid-lowering therapy. No significant adverse events were reported. Ketosis was observed in all patients, with β-Hydroxybutyrate capillary blood concentrations between 0.5–1 mmol/dL, and no significant differences were found between the two groups (data not shown).

### Changes in body mass index, body composition, and muscle strength

At the end of the study, we observed a significant reduction of total BW both in the VLCKD group (–9.6 ± 1.61 kg compared to baseline; average percentage BW loss: 10.5%), and in the VLCKD + IT (–10.4 ± 3.2 kg compared to baseline; average percentage BW loss: 11.4%).

Body mass index followed the same pattern, with a significant reduction both in the VLCKD group (–3.6 ± 0.6 kg/m^2^) and in the VLCKD + IT group (–4.0 ± 1.3 kg/m^2^). A reduction in WC and HC was observed in all groups and reached statistical significance as shown in Table 3.

**TABLE 3:** Participants characteristics at baseline (T0) and at the end of the study (T6).

	VLCKD				VLCKD + IT				*P*-value
	T0	T6	Δ	*p*	T0	T6	Δ	*p*	
Weight (kg)	90.5 ± 7.1	81.0 ± 6.1	–9.6 ± 1.61	<0.001	91.6 ± 12.1	81.1 ± 11.5	–10.4 ± 3.2	<0.001	0.451
WC (cm)	101.2 ± 9.7	92.3 ± 8.6	–8.9 ± 3.5	**<0.001**	100.7 ± 8.3	91.7 ± 8.1	–9.1 ± 2.5	**<0.001**	0.895
HC (cm)	122.7 ± 9.2	115.2 ± 9.6	–7.5 ± 1.4	**<0.001**	123.0 ± 10.1	113.0 ± 10.5	–10.0 ± 3.0	**<0.001**	**0.025**
FM (kg)	40.53 ± 11.63	33.97 ± 10.94	–6.5 ± 1.9	**<0.001**	39.59 ± 8.62	28.69 ± 8.67	–11.0 ± 3.4	**<0.001**	**0.001**
FFM (kg)	52.62 ± 5.42	50.32 ± 5.46	–2.3 ± 1.3	**<0.001**	52.16 ± 5.04	52.46 ± 5.01	0.3 ± 1.0	0.329	**<0.001**
BMI (kg/m^2^)	33.8 ± 3.0	30.2 ± 2.6	–3.6 ± 0.6	**<0.001**	33.9 ± 3.8	29.9 ± 3.5	–4.0 ± 1.3	**<0.001**	0.389
BMR (kcal)	1513.75 ± 100.92	1455.58 ± 93.16	–58.1 ± 35.0	**<0.001**	1491.16 ± 106.75	1499.41 ± 105.85	8.2 ± 22.1	0.224	**<0.001**
BCM (kg)	30.39 ± 4.84	26.56 ± 4.09	–3.8 ± 3.5	**0.003**	27.65 ± 3.11	29.34 ± 6.85	1.6 ± 4.5	0.226	**0.003**

Variables with normal distribution are expressed as mean ± SD, those with non-normal distribution as median (interquartile range). VLCKD, very low calorie ketogenic diet; IT, interval training; WC, waist circumference; HC, hip circumference; FM, fat mass; FFM, fat free mass; BMI, body mass index; BMR, basal metabolic rate; BCM, body cell mass. The bold represents statistically significant values.

**FIGURE 2:**
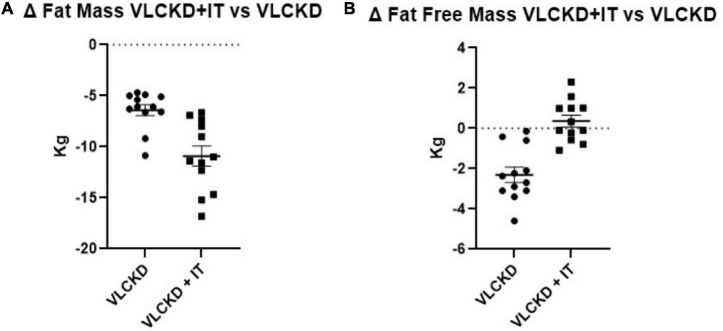
Grouped ranking charts of observed relative differences (% variation vs. basal values) from T0 to T6 in fat mass **(A)** and fat free mass **(B)** in VLCKD and VLCKD + IT groups. *P*-values of the parameters plotted are shown in Table 1.

Fat mass was reduced in both groups (VLCKD: –6.5 ± 1.9 kg compared to baseline; VLCDK + IT: –11.0 ± 3.4 kg compared to baseline) but at a significantly higher extent more in the VLCKD + IT group than in the VLCKD group (Figure 2). Notably, FFM was slightly reduced in the VLCKD group (–2.3 ± 1.3 kg compared to baseline) while it was not altered in the VLCKD + IT group (0.3 ± 1.0 Kg compared to baseline, *p* = 0.329) as shown in Figure 2.

In parallel, a significant reduction in BMR, estimated by BIA, was observed only in the VLCKD group (*p* < 0.001), in keeping with FFM data. Of note, the VLCKD + IT group did not show any statistically significant change in BCM and BMR (*p* = 0.224 and *p* = 0.226, respectively).

Muscle strength, measured through the CST, was increased in both groups (*p* < 0.001), with no significant differences between the two groups (*p* = 0.483) (Figure 2). No significant differences were detected on physical performance, as measured through SPPB (data not shown).

Univariate and multiple linear regression analyses were used to evaluate associations between Δ BMR and Δ BCM respect to the change in FFM and FM into group, adjusting for age and sex. As shown in Table 4, the models generated by the multiple linear regression analysis to estimate Δ BMR and Δ BCM showed differences regarding the covariates (FFM and FM).

**TABLE 4:** Univariate linear regression.

Outcome variables	Variables	Groups	β ± SE	CI 95%		*P*
				Lower limit High limit		
Δ BMR Model 1	Δ FFM	VLCKD^a^ VLCKD + IT^b^	13.98 ± 5.28 19.39 ± 3.06	1.80 12.32	26.15 26.45	**0.029** **<0.001**
Δ BMR Model 2	Δ FM	VLCKD^c^ VLCKD + IT^d^	–3.91 ± 4.88 –0.66 ± 1.88	−15.18 −5.01	7.36 3.69	0.447 0.736
Δ BCM Model 1	Δ FFM	VLCKD^e^ VLCKD + IT^f^	1.45 ± 0.63 0.60 ± 1.82	0.00 −3.60	2.90 4.79	**0.05** 0.751
Δ BCM Model 2	Δ FM	VLCKD^g^ VLCKD + IT^h^	–0.85 ± 0.48 –0.92 ± 0.33	−1.97 −1.68	0.27 −0.15	0.118 **0.024**

Primary outcomes are Δ BMR and Δ BCM; variables selected are FFM and FM, adjusted for sex and age. ^a^R^2 adjusted 0.59. ^b^R^2 adjusted 0.85. ^c^R^2 adjusted 0.29. ^d^R^2 adjusted 0.10. ^e^R^2 adjusted 0.10. ^f^R^2 adjusted 0.07. ^g^R^2 adjusted 0.31. ^h^R^2 adjusted 0.34. SE, standard errors; CI, confidence interval; BMR, basal metabolic rate; FFM, fat free mass; VLCKD, very low calorie ketogenic diet; IT, interval training; FM, fat mass; BCM, body cell mass. The bold represents statistically significant values.

Notably, in model 1, Δ BMR was found positively associated with Δ FFM with a high value for β in both VLCKD (β ± SE: 13.98 ± 5.28; *p* = 0.029) and VLCKD + IT groups (β ± SE: 19.39 ± 3.06; *p* < 0.001), indicating that an increase of Δ BMR (19.39 ± 3.06 Kcal) takes place in response to the unitary variation in FFM.

Moreover, in model 2, Δ BCM was found negatively associated with Δ FM (β ± SE: –0.92 ± 0.33; *p* = 0.024), indicating that an increase in Δ BCM takes place in response to the unitary variation in FM (Table 4), only in the VLCKD + IT group.

Multiple regression analysis confirmed the relationship between Δ BMR with Δ FFM only in VLCKD + IT group (β ± SE: 19.67 ± 3.30; *p* = 0.001), as shown in Table 5. The regression model predicted 83% of the variance. The model was suitable for predicting the outcome (F = 14.438, df = 11, *p* = 0.002).

**TABLE 5:** Multiple linear regression analysis of BMR and BCM with two variables combined (D FM and D FFM), adjusted sex and age.

Outcome variables	Variables	Groups	β ± SE	CI 95%		*P*
				Lower limit High limit		
Δ BMR	Δ FFM	VLCKD^a^	15.71 ± 6.67	−0.07	31.49	0.051
		VLCKD + IT^c^	19.67 ± 3.30	11.86	27.47	0.001
	Δ FM	VLCKD	2.19 ± 4.69	−8.89	13.28	0.654
		VLCKD + IT	0.35 ± 0.84	−1.63	2.33	0.688
Δ BCM	Δ FFM	VLCKD^b^	1.13 ± 0.78	−0.71	2.96	0.190
		VLCKD + IT^d^	–0.14 ± 1.43	−3.52	3.23	0.923
	Δ FM	VLCKD	–0.41 ± 0.54	−1.70	0.88	0.475
		VLCKD + IT	–0.93 ± 0.36	−1.78	−0.071	**0.037**

^a^R^^2^ Adjusted 0.54. ^b^R^^2^ Adjusted 0.39. ^c^R^^2^ Adjusted 0.83. ^d^R^^2^ Adjusted 0.25. SE, standard errors; CI, confidence interval; BMR, basal metabolic rate; FFM, fat free mass; VLCKD, very low calorie ketogenic diet; IT, interval training; FM, fat mass; BCM, body cell mass. The bold represents statistically significant values.

Moreover, multiple regression analysis confirmed the relationship between Δ BCM with Δ FM only in the VLCKD + IT group (β ± SE: –0.93 ± 0.36; *p* = 0.037). The regression model predicted 25% of the variance. The model was not suitable for predicting the outcome (F = 1.903, df = 11, *p* = 0.215).

### Change in biochemical parameters

A significant reduction in fasting glycemia, fasting insulin, and HOMA-IR was observed in all groups (Table 6). Electrolytes (data not shown) did not change within groups during the study. A significant reduction in BUN was found in the VLCKD group (*p* = 0.002), while only a trend of reduction was observed in the VLCKD + IT group. A significant reduction in creatinine was found in both VLCKD and VLCKD + IT groups. An improvement in eGFR was found only in the VLCKD + IT group (*p* = 0.013).

**TABLE 6:** Participants characteristics at baseline (T0) and at the end of the study (T6).

	VLCKD				VLCKD + IT				*P*-value
	T0	T6	Δ	*p*	T0	T6	Δ	*p*	
Fasting glycemia (mg/dL)	105.9 ± 12.8	86.6 ± 12.5	–16.3 ± 9.5	**<0.001**	101.4 ± 11.7	87.0 ± 8.6	–14.4 ± 8.4	**<0.001**	0.606
Fasting insulin (μIU/ml)	11.8 (11.0–13.75)	8.1 (7.25–9.75)	–	0.002*	12.7 (11.25–14.57)	9.0 (8.3–9.4)	–	0.002*	0.630^+^
HOMA-IR	3.9 ± 2.9	1.8 ± 0.6	–2.1 ± 2.6	**0.018**	3.3 ± 0.8	1.8 ± 0.4	–1.5 ± 0.6	**<0.001**	0.474
BUN (mg/dl)	37.3 ± 7.8	32.3 ± 5.6	–5.0 ± 4.3	**0.002**	34.1 ± 9.1	30.4 ± 5.9	–3.7 ± 6.5	0.075	0.579
Creatinine (mg/dl)	0.82 ± 0.18	0.78 ± 0.16	–0.04 ± 0.06	**0.046**	0.80 ± 0.13	0.72 ± 0.08	–0.08 ± 0.09	**0.017**	0.276
eGFR (ml/min)	83.87 ± 15.51	88.84 ± 15.21	4.97 ± 9.74	0.105	82.50 ± 14.84	91.69 ± 11.05	9.18 ± 10.78	**0.013**	0.327
AST (U/L)	23.5 ± 5.7	19.8 ± 5.3	–3.6 ± 4.9	**0.026**	32.3 ± 12.9	24.6 ± 6.7	–7.7 ± 10.9	**0.032**	0.251
ALT (U/L)	26.0 ± 11.1	19.1 ± 6.7	–6.8 ± 8.2	**0.015**	37.8 ± 14.6	26.3 ± 12.1	–11.5 ± 11.5	**0.005**	0.267
Total cholesterol (mg/dl)	203.7 ± 38.6	161.8 ± 30.5	–41.9 ± 20.1	**<0.001**	212.8 ± 23.6	185.0 ± 17.3	–27.8 ± 23.9	**0.002**	0.133
HDL cholesterol (mg/dl)	49.5 (41.2–63.7)	51.5 (40.0–63.0)	–1.0 (–6.5; 2.0)	0.431*	47.5 (42.5–52.2)	53.0 (50.5–64.0)	6.0 (1.25; 9.75)	0.003*	**0.002^+^**
LDL cholesterol (mg/dl)	127.5 ± 36.7	93.8 ± 23.8	–33.7 ± 24.5	**0.001**	139.9 ± 31.0	110.9 ± 18.5	–29.0 ± 29.9	**0.006**	0.678
Triglycerides (mg/dl)	132.8 ± 38.1	83.5 ± 16.9	–49.3 ± 30.0	**<0.001**	114.9 ± 25.2	80.4 ± 12.2	–34.5 ± 24.6	**0.001**	0.199
Vitamin D (ng/dl)	18.1 ± 8.3	21.1 ± 9.6	2.9 ± 2.5	**0.002**	24.8 ± 11.4	31.0 ± 9.7	6.1 ± 5.4	**0.003**	0.080

Variables with normal distribution are expressed as mean ± SD, those with non-normal distribution as median (interquartile range) with Wilcoxon test. VLCKD, very low calorie ketogenic diet; IT, interval training; HOMA-IR, homeostasis model assessment-insulin resistance; BUN, blood urea nitrogen; eGFR, glomerular filtration rate; AST, aspartate aminotransferase; ALT, alanine aminotransferase; HDL, high density lipoprotein; LDL, low density lipoprotein. *Wilcoxon test; ^+^Mann Whitney. The bold represents statistically significant values.

An improvement in lipid profile was observed in both groups, as shown in Table 6; importantly, HDL plasma levels were significantly increased in the VLCKD + IT group compared to the VLCKD group.

## Discussion

To date, there are still no clear indications regarding the type of physical activity to be performed during a VLCKD. In some cases, healthcare providers even suggest that the patient starting a VLCKD protocol should stop physical activity, due to the low calorie and carbohydrate content of the nutritional treatment.

**FIGURE 3:**
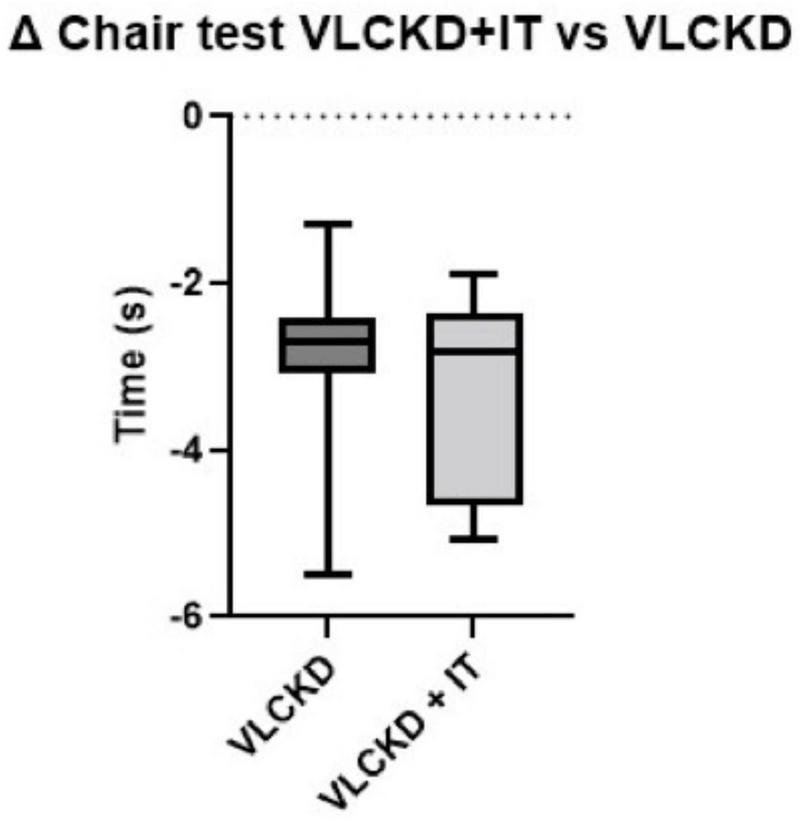
Box plot of pooled ranking of observed relative differences (% variation vs. basal values) from T0 to T6 in Chair stand test in VLCKD and VLCKD + IT groups.

In this study, we demonstrated that a VLCKD combined with physical IT from the very beginning of the protocol improved body composition by reducing FM and preserving FFM to a greater extent than VLCKD alone. Importantly, patients following VLCKD showed a significant increase in muscle strength and function after 6-weeks of treatment, independently of IT, as shown in Figure 3. As shown in a recent study by Romano et al. (24), a reduction in FFM, more specifically in lean mass, has been observed during VLCKD in patients with type 2 diabetes, but this variation was limited to the beginning of the nutritional protocol, whereas, only a loss of FM was observed subsequently. In keeping with this, as observed in a previous study by Merra et al. (25), VLCKD was effective as a dietary approach determining a reduction in BW, FM and FFM maintenance, in patients with obesity. In general, skeletal muscle loss may occur in the context of BW reduction during a hypocaloric diet (26), and an increase in muscle proteolysis has been suggested to play a role in muscle mass reduction under calorie restriction. Data obtained in these studies confirm that VLCKD, albeit a slight reduction in initial lean mass, results in a preservation of skeletal mass. In addition, our study evaluated the role of physical training during a VLCKD protocol. These results, although preliminary and observed in a limited number of patients, confirm that it is essential to combine physical training during the VLCKD protocol in order to maintain FFM. Furthermore, the VLCKD + IT group did not experience any significant reduction in BMR and BCM, and the preservation of FFM, despite the caloric deficit, was determined by physical training.

In both groups, physical performance was significantly increased, irrespective of physical training. We speculate that the improvement in physical performance was mainly explained by the relevant weight loss in both groups, together with the improved nutritional and inflammatory status. Of note, we failed to observe an improvement of physical performance in the VLCKD IT group. It is possible that the tests used in this study were not the most appropriate to highlight potential advantages due to physical training.

It is well-known, as reported in a study by Frank et al. (27), that physical activity is directly related to cardiometabolic health. In fact, 150 min of physical activity have a positive impact on reducing BMI and increasing HDL plasma levels. Remarkably, we did observe a significant increase of HDL plasma levels only in the VLCKD-IT group (*p* = 0.002). This was not observed in patients following VLCKD alone.

In our study, a significant improvement in all metabolic parameters was observed, independently of the physical training.

In addition, an increase in vitamin D levels was found in both VLCKD and VLCKD + IT groups (*p* = 0.002 and *p* = 0.003, respectively). As already reported in many studies, subjects with obesity have low vitamin D levels, as it is sequestered in fat cells (28, 29). Whenever a reduction in BW and FM occurs, there is an upward trend in Vitamin D levels, with favorable effects of metabolic and endothelial function (30).

The VLCKD was safe and well-tolerated for 6-weeks; in fact, a significant reduction in BUN and creatinine was observed in both groups, together with a mild improvement in eGFR. These results, confirm that VLCKD is safe and does not lead to a worsening of renal function in patients with visceral obesity and sarcopenia.

However, we still remind that VLCKD is a nutritional approach requiring strict medical surveillance. Our pilot study has several limitations: first of all the number of participants enrolled was small, although sufficient to appreciate the changes induced by VLCKD. The short duration of the study protocol represents a further limitation, together with the lack of follow-up. Furthermore, due to the pandemic, physical training was performed *via* the Zoom platform: in-person exercise would certainly have been even more accurate in terms of control of the quality of exercise performance. Body composition analysis was performed by BIA, and not by DXA. Finally, a further limitation of our study is represented by the lack of individualized training intensity; in fact, only Borg’s RPE scale was used at the end of each training session to test the perception of fatigue (31, 32).

## Conclusion

Evidence supporting the beneficial impact of VLCKD on adipose and skeletal muscle metabolism in the management of obesity is increasing. In patients with obesity, this dietary approach markedly improved lipid and glycemic profiles, with proven cardiometabolic protective effects. This pilot study shows for the first time that VLCKD combined with physical interval training reduces adipose depots and preserves FFM, with a preservation of muscle strength during weight loss and an increase in plasma HDL cholesterol. Of course, these data need to be confirmed in a larger population, in order to draw solid conclusions on these relevant issues.

## Data availability statement

The original contributions presented in this study are included in the article/supplementary material, further inquiries can be directed to the corresponding author.

## Ethics statement

The studies involving human participants were reviewed and approved by Ethics Committee of the University of Rome “La Sapienza.” The patients/participants provided their written informed consent to participate in this study.

## Author contributions

EC, SB, and MC: study concept and design. EC and MC: acquisition of data. EC, AF, SP, and MC: interpretation of the data. EC, AF, ML, SB, and MC: analysis. EC, AF, AA, LB, LG, and MC: writing of the manuscript. All authors approved the final version of the manuscript.
