# Corrigendum: Very low calorie ketogenic diet combined with physical interval training for preserving muscle mass during weight loss in sarcopenic obesity: A pilot study

**DOI:** 10.3389/fnut.2022.1076667

**Published:** 2022-11-08

**Authors:** Elisabetta Camajani, Alessandra Feraco, Stefania Proietti, Sabrina Basciani, Luigi Barrea, Andrea Armani, Mauro Lombardo, Lucio Gnessi, Massimiliano Caprio

**Affiliations:** ^1^Department of Experimental Medicine, Sapienza University of Rome, Rome, Italy; ^2^Department of Human Sciences and Promotion of the Quality of Life, San Raffaele Roma Open University, Rome, Italy; ^3^Laboratory of Cardiovascular Endocrinology, Istituto di Ricovero e Cura a Carattere Scientifico (IRCCS) San Raffaele, Rome, Italy; ^4^Unit of Clinical and Molecular Epidemiology, Istituto di Ricovero e Cura a Carattere Scientifico (IRCCS) San Raffaele, Rome, Italy; ^5^Dipartimento di Scienze Umanistiche, Università Telematica Pegaso, Naples, Italy; ^6^Department of Clinical Medicine and Surgery, Endocrinology Unit, Centro Italiano per la cura e il Benessere del Paziente con Obesità, University Medical School of Naples, Naples, Italy

**Keywords:** VLCKD, sarcopenia, physical activity, fat free mass, fat mass

In the published article, there was an error in the **Funding** statement. A source of funding was missing from the statement (Italian Ministry of Health). The correct **Funding** statement appears below.

## Funding

This manuscript was funded by the MIUR (Progetti di Ricerca di Interesse Nazionale 2017—Project code 2017A5TXC3—to MC, Work Package Leader), by Institutional Fundings of the Ph.D. Program in Endocrinological Sciences, Sapienza University of Rome, and by funding of the Italian Ministry of Health (Ricerca Corrente). Meal replacement protein preparations were kindly provided by Dieta Medicale (Italy). The funding source had no involvement in the study design, study interventions, data collection, or interpretation of the results.

The authors apologize for this error and state that this does not change the scientific conclusions of the article in any way. The original article has been updated.

## Publisher's note

All claims expressed in this article are solely those of the authors and do not necessarily represent those of their affiliated organizations, or those of the publisher, the editors and the reviewers. Any product that may be evaluated in this article, or claim that may be made by its manufacturer, is not guaranteed or endorsed by the publisher.

